# Molecular and cellular characteristics of hybrid vigour in a commercial hybrid of Chinese cabbage

**DOI:** 10.1186/s12870-016-0734-3

**Published:** 2016-02-17

**Authors:** Natsumi Saeki, Takahiro Kawanabe, Hua Ying, Motoki Shimizu, Mikiko Kojima, Hiroshi Abe, Keiichi Okazaki, Makoto Kaji, Jennifer M. Taylor, Hitoshi Sakakibara, W. James Peacock, Elizabeth S. Dennis, Ryo Fujimoto

**Affiliations:** Graduate School of Science and Technology, Niigata University, Ikarashi-ninocho, Niigata, 950-2181 Japan; Graduate School of Agricultural Science, Kobe University, Rokkodai, Nada-ku, Kobe, 657-8501 Japan; CSIRO Agriculture, Canberra, ACT 2601 Australia; Center for Sustainable Resource Science, 1-7-22, Suehiro, Tsurumi, Yokohama, 230-0045 Japan; Experimental Plant Division, RIKEN BioResource Center, Tsukuba, 305-0074 Japan; Watanabe Seed Co., Ltd, Machiyashiki, Misato-cho, Miyagi 987-0003 Japan; University of Technology, Broadway, Sydney, PO Box 123, NSW 2007 Australia; Japan Science and Technology Agency (JST), Precursory Research for Embryonic Science and Technology (PRESTO), Saitama, 332-0012 Japan

**Keywords:** Heterosis, Hybrid vigour, Yield, gene expression, Chloroplast-targeted genes, Chinese cabbage

## Abstract

**Background:**

Heterosis or hybrid vigour is a phenomenon in which hybrid progeny exhibit superior performance compared to their parental inbred lines. Most commercial Chinese cabbage cultivars are F_1_ hybrids and their level of hybrid vigour is of critical importance and is a key selection criterion in the breeding system.

**Results:**

We have characterized the heterotic phenotype of one F_1_ hybrid cultivar of Chinese cabbage and its parental lines from early- to late-developmental stages of the plants. Hybrid cotyledons are larger than those of the parents at 4 days after sowing and biomass in the hybrid, determined by the fresh weight of leaves, is greater than that of the larger parent line by approximately 20 % at 14 days after sowing. The final yield of the hybrid harvested at 63 days after sowing is 25 % greater than the yield of the better parent. The larger leaves of the hybrid are a consequence of increased cell size and number of the photosynthetic palisade mesophyll cells and other leaf cells. The accumulation of plant hormones in the F_1_ was within the range of the parental levels at both 2 and 10 days after sowing. Two days after sowing, the expression levels of chloroplast-targeted genes in the cotyledon cells were upregulated in the F_1_ hybrid relative to their mid parent values. Shutdown of chlorophyll biosynthesis in the cotyledon by norflurazon prevented the increased leaf area in the F_1_ hybrid.

**Conclusions:**

In the cotyledons of F_1_ hybrids, chloroplast-targeted genes were upregulated at 2 days after sowing. The increased activity levels of this group of genes suggested that their differential transcription levels could be important for establishing early heterosis but the increased transcription levels were transient. Inhibition of the photosynthetic process in the cotyledon reduced heterosis in later seedling stages. These observations suggest early developmental events in the germinating seedling of the hybrid may be important for later developmental vigour and yield advantage.

**Electronic supplementary material:**

The online version of this article (doi:10.1186/s12870-016-0734-3) contains supplementary material, which is available to authorized users.

## Background

Hybrid vigour or heterosis refers to the superior performance of hybrid progeny relative to their parents, and this phenomenon is important in the production of many crops and vegetables. Genetic analyses of F_1_ hybrids in maize and rice have defined a large number of QTLs, which may make contributions to heterosis. Gene interactions such as dominance, overdominance, pseudo-overdominance, and epistasis have been suggested to explain the development of heterosis [1, 2]. Recent molecular analyses of transcriptomes, proteomes, and metabolomes, together with reference to the epigenome of the parents and hybrids have begun to uncover some new facts about the generation of hybrid vigour [3–6]. High-throughput sequencing technology enables us to not only compare the expression level of genes between the F_1_ and parental lines but also to examine the parental allelic contributions to gene expression in F_1_ hybrids at the whole genome level [7].

In *Arabidopsis thaliana,* several hybrids such as Columbia-0 (Col) x C24 and Landsberg *erecta* (L*er*) x C24 show heterosis in vegetative biomass. A heterosis phenotype is seen in early development with hybrids having increased cotyledon size only a few days after sowing [8–11]. The efficiency of the photosynthetic process is equivalent in parents and C24 x Col hybrids, and leaves of the hybrids are larger than the leaves of the parents. The total amount of photosynthesis is greater in the hybrids than in parents because of the larger leaves [9].

The genus Brassica includes important vegetables (*Brassica rapa* L. and *Brassica oleracea* L.) and oilseed crops (*Brassica napus* L.), and is related to *A. thaliana. B. rapa* vegetables such as Chinese cabbage (var. *pekinensis*), turnip (var. *rapa*), pak choi (var. *chinensis*), and Komatsuna (var. *perviridis*) are widely grown in Asia. Most cultivars of *B. rapa* are self-incompatible, preventing self-fertilization, although some oilseed cultivars (var. *tricolaris*) are self-compatible [12–14]. In Japan, most *B. rapa* commercial varieties are F_1_ hybrid cultivars which have increased yields relative to their parents. Self-incompatibility or cytoplasmic male sterility is utilized in producing the F_1_ hybrid seeds [14].

Though there is no doubt that F_1_ hybrids exhibit heterosis in yield, there are few reports evaluating the yield characteristics of Chinese cabbage hybrids, and there is no report focusing on early developmental stages of the hybrid plant. In this study, we examined the plant size and hormone concentrations in early seedlings and yield in the commercial Chinese cabbage hybrid “W39” and its parents to find when heterosis occurs and how much the yield increases in the F_1_ hybrid relative to parental lines. It has been suggested that heterosis could be a result of changes in the transcriptional network. We identified the differentially expressed genes between the F_1_ and parental lines together with the allele-specific expressed genes in the F_1_ at 2 days after sowing (DAS) by RNA sequencing (RNA-seq). We found that increased production of photosynthesis in the first week after germination is critical for heterosis and that upregulation of chloroplast-targeted genes at 2 DAS might contribute to this process.

## Methods

### Plant materials

A commercial F_1_ hybrid cultivar of Chinese cabbage, “W39” (Watanabe Seed Co. Ltd., Japan), and its parental inbred lines, S27 (female) and R29 (male), were used for analysis of the heterosis phenotype. Selfed seeds of parental lines were harvested using honeybees as pollinators after spraying with NaCl solution, which weakens the self-incompatibility. Seeds of F_1_ hybrids were harvested by open crossing between parental lines. Fifty dry seeds of parental lines and hybrids were weighed and statistical comparisons of the weight of 50 dry seeds were performed using Student’s *t*-test (*p* < 0.05).

Plants were grown in plastic dishes containing Murashige and Skoog (MS) agar medium supplemented with 1.0 % sucrose (pH 5.7) in growth chambers under a 16-h/8-h light/dark cycle at 22 °C. The parents and hybrids were placed at equal intervals on the same agar plate divided into two or four regions (Additional file 1: Fig. S1A), and samples were harvested for examination of cotyledon/leaf area and cell size, flow cytometric analysis, hormonome analysis, chlorophyll quantification, and expression analysis.

For the inhibitor studies, seedlings were grown for a week on MS plates and transferred to MS plates with 1.0 μM norflurazon (Sigma-Aldrich), or seeds were sown on the MS plates with 1.0 μM norflurazon and after one week treated seedlings were transferred to MS plates.

For examining the yield under field conditions, seeds were sown on multi cell trays on 17th August 2011 and grown in a greenhouse. On 5th September 2011, seedlings were transplanted to the field at Osaki, Miyagi, Japan (38°57’N, 141°00’E). Thirty plants per plot were transplanted and plot size was 13.5 x 0.7 meters. Row spacing is 70 cm and planting distance is 40 cm. On 29th October 2011, plants were harvested. Statistical comparisons of fresh weight of total biomass and harvested biomass were performed using Student’s *t*-test (*p* < 0.05).

### Cotyledon/leaf area and cell size

Cotyledons in seeds, cotyledons at 2, 4, or 6 DAS, and 1st and 2nd leaves at 10, 12, or 14 DAS were fixed in a formalin/acetic acid/alcohol solution (ethanol: acetic acid: formalin = 16: 1: 1). The image of the whole cotyledon or leaf was photographed under a stereoscopic microscope, and sizes were determined with Image-J software (http://rsb.info.nih.gov/ij/). After examination of cotyledon or leaf area, they were cleared in a chloral hydrate/glycerol/water solution (chloral hydrate: H_2_O: glycerol = 8: 2: 1), and the samples were photographed under Nomarski optics. The palisade cell number per fixed unit area in the subepidermal layer of the center of the leaf blade between the midvein and the leaf margin was counted. More than three independent experiments were performed for examination of cotyledon/leaf area and cell size. Statistical comparisons of cotyledon/leaf area and cell size were performed using Student’s *t*-test (*p* < 0.05).

### Flow cytometric analysis

Nuclei from cotyledons at 6 DAS or 1st and 2nd leaves at 14 DAS grown on MS agar plates in a growth chamber were released in nuclei extraction buffer by lightly chopping the cotyledons or leaves with a razor blade and stained following the manual of Partec CyStain UV precise P (PARTEC). Ploidy levels were measured by a Ploidy Analyzer (PARTEC). Flow cytometry experiments were repeated three times using cotyledons or true leaves from different plants.

### Hormone analysis

The 2 day cotyledon and 10 day 1st and 2nd leaves were harvested. Plant hormones were extracted, purified, and quantified as described previously [15, 16]. Statistical comparisons of plant hormone contents were performed using Student’s *t*-test (*p* < 0.05).

### Chlorophyll extraction and quantification

Cotyledons at 6 DAS were ground in 80 % (vol/vol) acetone. Absorbance of the supernatants was measured at 646.6 and 663.6 nm, and concentrations of total chlorophyll were calculated using the following formulae: total chlorophyll (μg/mL) = 17.76 × A646.6 + 7.34 × A663.6. Data presented are the average and standard error (SE) from six biological replications.

### Gene expression analysis

The parents and hybrids were grown on MS agar plates in a growth chamber. Total RNA was isolated from five bulked cotyledons of both hybrids and parents from 2 – 6 DAS using the SV Total RNA Isolation System (Promega). cDNA was synthesized from 500 ng total RNA using PrimeScript RT reagent Kit (Takara bio). Prior to quantitative RT-PCR, the specificity of the primer set for each gene was first tested by electrophoresis of PCR amplified products using EmeraldAmp MAX PCR Master Mix (Takara bio) on 2.0 % agarose gel in which single products were observed. Absence of genomic DNA contamination was confirmed by the PCR of no RT control. PCR conditions were 95 °C for 3 min followed by 30 cycles of 95 °C for 30 s, 55 °C for 30 s, and 72 °C for 30 s.

Quantitative RT-PCR was performed using a LightCycler Nano (Roche). The cDNA was amplified using FastStart Essential DNA Green Master (Roche). PCR conditions were 95 °C for 10 min followed by 40 cycles of 95 °C for 10 s, 60 °C for 10 s, and 72 °C for 15 s, and Melting program (60 °C to 95 °C at 0.1 °C/s). After amplification cycles, each reaction was subjected to melt temperature analysis to confirm single amplified products. The relative expression level of each gene relative to *ACTIN* (*Bractin*) was automatically calculated using automatic CQ calling according to the manufacturer’s instructions (Roche) [17]. Data presented are the average and SE from three biological and experimental replications and statistically analysed using the Student’s *t*-test, *p* < 0.05. The primers used in this study are listed in Additional file 2: Table S1.

### RNA sequencing

Cotyledons were collected at 2 DAS and total RNA was isolated with SV Total RNA Isolation System (Promega). Sequence library preparation, sequencing, mapping short reads, identification of differentially expressed genes, and gene ontology analysis were followed as described previously [18]. RNA-seq was performed using Illumina Hiseq2000. Totally, 16,357,770 (~1500 Mbp), 17,548,397 (~1600 Mbp), and 16,267,428 (~1500 Mbp) reads in S27, R29, and F_1_ were uniquely mapped to *Brassica* genome release 1.2, respectively. The gene expression level was scored by fragments per kilobase per million (FPKM). The merged reads of S27 and R29 were used for mid-parent values (MPV).

We searched the SNPs between S27 and R29 from RNA-seq data with a minimum coverage of eight reads per site. Of 41,174 annotated genes, 10,931 genes (26.5 %) had no reads both in S27 and R29, and 12,770 (31.0 %) genes had more than one SNP.

## Results

### Heterosis can be detected in young seedlings

We followed the development of the leaves in the hybrid and parents from germination to 30 DAS. The germination rate did not differ among parental lines and F_1_ hybrids. The R29 parent had more leaves from 12 to 30 DAS than did the F_1_ hybrid or the S27 parent (Additional file 1: Fig. S1B). At 30 DAS the F_1_ hybrid had 71 % and 11 % greater fresh weight than the S27 and R29 parental lines, respectively (Additional file 1: Fig. S1C).

The mature seeds of the F_1_ hybrid have a greater dry weight than the parental seeds (Table 1), and the cotyledon in the mature F_1_ seed has an increased area relative to the area of the cotyledon in the better performing parent R29 (Table 1). We checked whether the increased size of the F_1_ cotyledon was due to an increased number or to increased size of the palisade mesophyll cells in the cotyledon, or whether both factors apply. The adaxial layer of palisade mesophyll cells has fewer cells per unit area in the F_1_ hybrid than in the parental lines (Table 1), indicating the palisade cells are larger in the F_1_ hybrid than in the parents.

**Table 1:** Dry weight, cotyledon size, and cell number per unit area of cotyledon in mature seeds

	S27 (female)	R29 (male)	F_1_-S27 × R29
50 seed weight (mg)	138.3 ± 1.0^a^ (*n* = 5)	160.4 ± 1.3^b^ (*n* = 5)	171.9 ± 4.6^c^ (*n* = 5)
Cotyledon area (mm^2^)*	2.72 ± 0.08^a^ (*n* = 30)	3.04 ± 0.08^b^ (*n* = 30)	3.27 ± 0.08^c^ (*n* = 30)
Cell number per unit area (250 μm^2^)	55.09 ± 1.89^b^ (*n* = 11)	61.91 ± 1.48^c^ (*n* = 11)	47.77 ± 1.25^a^ (*n* = 13)

Different letters indicate significant differences at *p* < 0.05 (Student’s t-test)

*The area is half of the cotyledon

Mean ± Standard errors

In the germinating seedlings the cotyledons of the F_1_ hybrids remained larger than the cotyledons of the parents over the period 2–6 DAS (Table 2). The cotyledons begin to senescence after this time. The first two leaves of the F_1_ hybrid at 14 DAS were larger and wider than those of the larger parent, S27 (Table 2). The cotyledons and leaves of the F_1_ hybrids had cell sizes equal to the R29 parent, which has larger cells than the S27 parent (Table 3). The distribution of ploidy levels in the cells of the cotyledons and leaves in parents and the F_1_ hybrid showed no difference in the cotyledons at 6 DAS and 1st and 2nd leaves at 14 DAS (Additional file 1: Fig. S2). In seedling development the F_1_ hybrid had a greater fresh weight at 7 and 14 DAS than the larger parent (Table 2). Heterosis was not evident in the root system at either 7 or 14 DAS (data not shown).

**Table 2:** Area and size of cotyledon and true leaf and fresh weight in S27, R29, and F_1_

	S27 (female)	R29 (male)	F_1_-S27 × R29	Relative to MPV	Relative to BPV
Cotyledon area (mm^2^)					
2 DAS	10.77 ± 0.59^a^ (*n* = 19)	11.69 ± 0.48^ab^ (*n* = 20)	13.04 ± 0.68^b^ (*n* = 24)	1.16	1.12
4 DAS	52.87 ± 2.46^a^ (*n* = 42)	53.59 ± 2.14^a^ (*n* = 36)	68.82 ± 3.00^b^ (*n* = 33)	1.29	1.28
6 DAS	80.14 ± 3.51^a^ (*n* = 37)	77.13 ± 2.75^a^ (*n* = 38)	100.52 ± 4.69^b^ (*n* = 36)	1.28	1.25
1st and 2nd leaf area (mm^2^)					
10 DAS	87.62 ± 5.57^a^ (*n* = 26)	84.51 ± 2.03^a^ (*n* = 36)	119.26 ± 5.25^b^ (*n* = 30)	1.39	1.36
12 DAS	143.51 ± 5.59^b^ (*n* = 30)	111.01 ± 2.86^a^ (*n* = 36)	158.40 ± 6.57^b^ (*n* = 36)	1.24	1.10
14 DAS	146.48 ± 5.02^b ^(*n* = 36)	114.62 ± 4.62^a^ (*n* = 34)	179.13 ± 6.96^c^ (*n* = 36)	1.37	1.22
Leaf size at 14 DAS					
Length (cm)	1.50 ± 0.03^a^ (*n* = 36)	1.45 ± 0.03^a^ (*n* = 36)	1.74 ± 0.04^b^ (*n* = 36)	1.18	1.16
Width (cm)	0.94 ± 0.02^a^ (*n* = 36)	0.86 ± 0.02^a^ (*n* = 36)	1.13 ± 0.03^b^ (*n* = 36)	1.26	1.20
Fresh weight (mg)					
7 DAS	79.04 ± 1.88^a^ (*n* = 25)	77.51 ± 1.88^a^ (*n* = 37)	90.22 ± 2.32^b^ (*n* = 41)	1.15	1.14
14 DAS	239.86 ± 5.83^b^ (*n* = 42)	182.75 ± 4.24^a^ (*n* = 44)	257.63 ± 5.24^c^ (*n* = 43)	1.22	1.07

Letters indicate significant differences at *p* < 0.05 (Student’s t-test)

*MPV* mid-parent value, *BPV* best-parent value

Mean ± Standard errors

**Table 3:** Cell number per unit area in the first layer of palisade mesophyll cells of cotyledon and true leaf

	S27 (female)	R29 (male)	F_1_-S27 × R29
Cell number per unit area (400x400 μm^2^)			
Cotyledon at 2 DAS	2130.82 ± 81.54^a^ (*n* = 17)	1991.53 ± 52.32^a^ (*n* = 17)	1930.67 ± 55.06^a^ (*n* = 18)
Cotyledon at 4 DAS	152.12 ± 4.21^c^ (*n* = 17)	128.20 ± 5.75^b^ (*n* = 20)	111.63 ± 4.04^a^ (*n* = 19)
Cotyledon at 6 DAS	111.16 ± 4.67^b^ (*n* = 19)	86.35 ± 2.64^a^ (*n* = 20)	86.85 ± 3.15^a^ (*n* = 20)
Cell number per unit area (200x200 μm^2^)			
1st and 2nd leaves at 10 DAS	186.59 ± 6.75^b^ (*n* = 17)	109.82 ± 3.83^a^ (*n* = 17)	121.81 ± 4.92^a^ (*n* = 16)
1st and 2nd leaves at 12 DAS	119.61 ± 2.67^b^ (*n* = 18)	75.78 ± 2.39^a^ (*n* = 23)	82.06 ± 3.49^a^ (*n* = 17)
1st and 2nd leaves at 14 DAS	81.32 ± 3.80^b^ (*n* = 19)	66.74 ± 2.25^a^ (*n* = 19)	62.17 ± 4.02^a^ (*n* = 18)

Letters indicate significant differences at *p* < 0.05 (Student’s t-test)

Mean ± Standard errors

**Fig. 1:**
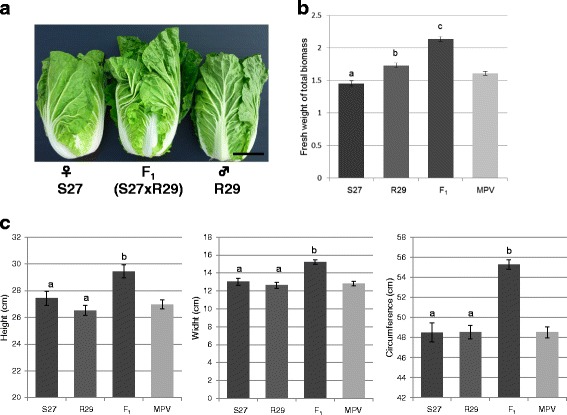
Harvested and total biomass of F_1_ hybrid and parents in Chinese cabbage. **a** Harvested biomass. The scale bar is 10 cm. **b** Fresh weight of total biomass in S27 (*n* = 23), R29 (*n* = 30), and F_1_ hybrid (*n* = 30). **c** Height, width, and circumference of harvested S27 (*n* = 15), R29 (*n* = 15), and F_1_ hybrid (*n* = 15). Letters above the bars indicate significant differences at *p* < 0.05 (Students *t*-test). MPV, mid parent value

In field conditions the F_1_ hybrid showed more than 20 % greater total biomass and harvested biomass (in which the outer leaves were stripped for marketing) than the larger parent (Fig. 1a, b). The height, width, and circumference of the harvested F_1_ plants were all greater than the corresponding dimensions of the parental plants (Fig. 1c).

### Hormone profiles were similar in parental lines and the F_1_ hybrid

**Fig. 2:**
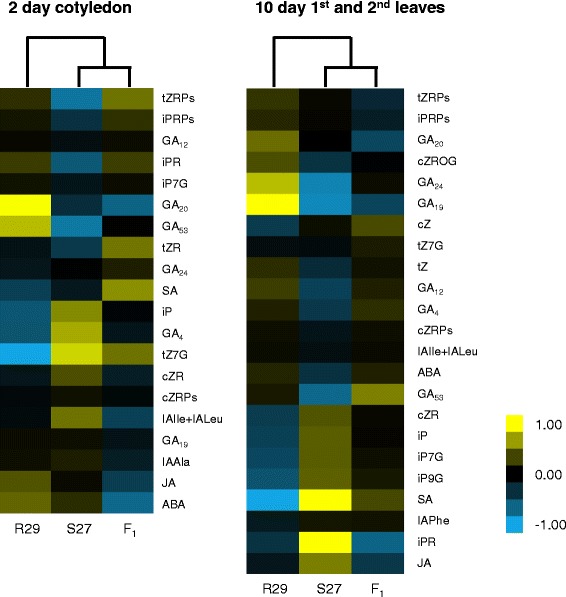
Hierarchical average linkage clustering of plant hormone contents. Hormone contents higher or lower than the median are shown in yellow and blue, respectively

As hormone signaling has been suggested to be important in heterotic hybrids of *A. thaliana* [19], we examined endogenous hormone contents in the parents and F_1_ hybrid. We measured the levels of auxins, cytokinins, ABA, gibberellins, jasmonates, and salicylic acid in 2 day cotyledons and 10 day 1st and 2nd leaves. 20 of the 43 hormone derivatives assayed were not detected in any lines (Additional file 2: Table S2). GA_8_ was not detected in F_1_ hybrid, tZ was not detected in S27 and R29, and IAPhe was not detected in R29 and the F_1_ hybrid. 10, 5, and 7 molecular types showed significantly different contents between S27 and R29, between S27 and F_1_ hybrid, and between R29 and F_1_ hybrid (*p* < 0.05). GA_8_, GA_12_, GA_20_, and GA_53_ accumulated to higher levels in R29 than in S27 and F_1_ hybrid (Fig. 2, Additional file 2: Table S2). SA had higher levels in the F_1_ hybrid than in the parents.

In the 10 day 1st and 2nd leaves, 15 of the 43 hormone types were not detected in any lines (Additional file 2: Table S2). As was the case in 2 day cotyledons, plant hormone accumulation did not show over or under dominance in the F_1_ hybrid except for iPRPs and GA_20_ (Additional file 2: Table S2). These results indicate that the accumulation of plant hormones in the F_1_ hybrid was within the range of the parental levels at both 2 and 10 DAS.

### Expression level of organ size-associated genes

**Fig. 3:**
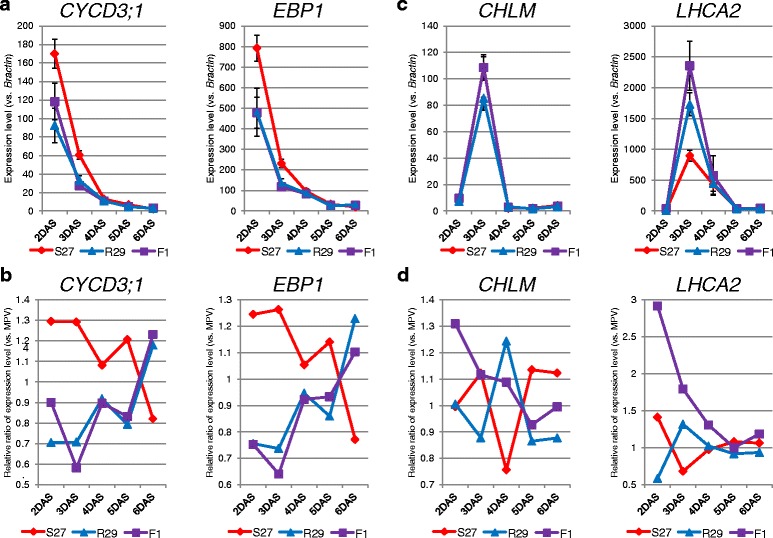
Expression level of genes involved in organ size (**a**, **b**) and chloroplast-targeted genes (**c**, **d**) in S27, R29, and F_1_ hybrid from 2 to 6 DAS. (**a**, **c**) The expression level compared with that of *Bractin.* (**b**, **d**) The relative expression level compared with MPV. Data is shown in Table 4

The increased cotyledon and leaf area in F_1_ hybrids suggested that organ size-associated genes contribute to the heterosis phenotype as has been reported in maize and *Larix* [20, 21]. We examined the expression level of four genes, *ARGOS*, *ANT*, *EBP1*, and *CYCD3;1*, which are involved in development of organ size [22], from 2 to 6 DAS and compared the expression levels between the F_1_ hybrid and each parent or between the F_1_ hybrid and MPV. The expression level of *ANT* was low, and the expression levels of *ARGOS*, *CYCD3;1*, and *EBP1* gradually decreased over time (Fig. 3a, b, Table 4). At 2 or 3 DAS, the expression levels of *ARGOS*, *CYCD3;1*, and *EBP1* in S27 were higher than those in R29 and F_1_ hybrids (Fig. 3a, b, Table 4), suggesting these loci do not contribute significantly to the heterosis of the F_1_ hybrid.

**Table 4:** Expression level of genes involved in organ size and chloroplast-targeted genes detected by quantitative RT-PCR at different times after sowing

	2 DAS		
	S27 (female)	R29 (male)	F_1_-S27 × R29
Genes involved in organ size			
* ANT*	0.05 ± 0.013^b^ (1.60)	0.02 ± 0.002^a^ (0.55)	0.03 ± 0.007^ab^ (1.11)
* CYCD3;1*	169.99 ± 15.920^b^ (1.29)	92.64 ± 18.702^a^ (0.71)	118.23 ± 19.826^ab^ (0.90)
* EBP1*	791.80 ± 63.295^b^ (1.24)	480.63 ± 117.658^ab^ (0.76)	478.61 ± 75.656^a^ (0.75)
* ARGOS*	38.14 ± 3.839^b^ (1.52)	12.16 ± 0.804^a^ (0.48)	18.60 ± 3.388^a^ (0.74)
Chloroplast-targeted genes			
* CHLM*	7.27 ± 0.723^a^ (1.00)	7.43 ± 0.849^a^ (1.00)	9.57 ± 0.116^b^ (1.31*)
* CHL27*	4.76 ± 0.368^ab^ (1.16)	3.47 ± 0.301^a^ (0.84)	5.94 ± 0.668^b^ (1.44*)
* PORB*	18.44 ± 2.558^b^ (1.46)	6.74 ± 1.238^a^ (0.54)	20.63 ± 2.589^b^ (1.64)
* LHCA2*	18.40 ± 0.671^b^ (1.41)	7.63 ± 0.691^a^ (0.59)	37.94 ± 5.624^c^ (2.91**)
* PORC*	1.22 ± 0.085^a^ (0.88)	1.54 ± 0.249^ab^ (1.12)	2.11 ± 0.107^b^ (1.53**)
* PsbS*	0.01 ± 0.001^a^ (0.86)	0.01 ± 0.005^a^ (1.14)	0.02 ± 0.008^a^ (2.21)
* ATPD*	17.06 ± 3.687^a^ (0.95)	18.89 ± 3.689^a^ (1.05)	39.26 ± 8.350^b^ (2.18*)
* PsbP*	54.56 ± 3.150^a^ (1.18)	37.57 ± 6.258^a^ (0.82)	96.10 ± 4.271^b^ (2.09**)
	3 DAS		
	S27 (female)	R29 (male)	F_1_-S27 × R29
Genes involved in organ size			
* ANT*	0.11 ± 0.035^a^ (1.47)	0.04 ± 0.009^a^ (0.53)	0.05 ± 0.024^a^ (0.72)
* CYCD3;1*	60.47 ± 4.589^b^ (1.29)	33.15 ± 5.176^a^ (0.71)	27.29 ± 4.522^a^ (0.58)
* EBP1*	231.00 ± 20.657^b^ (1.26)	134.80 ± 20.743^a^ (0.74)	117.18 ± 17.538^a^ (0.64)
* ARGOS*	11.76 ± 0.966^b^ (1.14)	8.86 ± 1.563^ab^ (0.86)	5.27 ± 0.442^a^ (0.51*)
Chloroplast-targeted genes			
* CHLM*	109.15 ± 7.716^a^ (1.12)	85.37 ± 9.127^a^ (0.88)	108.53 ± 9.602^a^ (1.12)
* CHL27*	273.81 ± 5.100^a^ (0.72)	484.32 ± 146.105^a^ (1.28)	223.76 ± 23.998^a^ (0.59)
* PORB*	879.58 ± 53.892^b^ (1.32)	456.81 ± 71.574^a^ (0.52)	509.17 ± 72.665^a^ (0.76)
* LHCA2*	895.36 ± 89.621^a^ (0.68)	1729.73 ± 188.985^ab^ (1.32)	2354.13 ± 398.359^b^ (1.79*)
* PORC*	34.55 ± 0.321^a^ (0.89)	43.16 ± 4.084^a^ (1.11)	41.58 ± 5.773^a^ (1.07)
* PsbS*	0.29 ± 0.011^a^ (0.90)	0.36 ± 0.041^a^ (1.10)	0.35 ± 0.046^a^ (1.06)
* ATPD*	1019.51 ± 74.779^a^ (1.03)	955.55 ± 121.680^a^ (0.97)	814.55 ± 216.658^a^ (0.82)
* PsbP*	3939.02 ± 403.225^a^ (0.94)	4445.87 ± 611.073^a^ (1.06)	6319.69 ± 1259.573^a^ (1.51)
	4 DAS		
	S27 (female)	R29 (male)	F_1_-S27 × R29
Genes involved in organ size			
* ANT*	0.03 ± 0.007^a^ (1.31)	0.02 ± 0.001^a^ (0.69)	0.03 ± 0.006^a^ (1.14)
* CYCD3;1*	12.91 ± 2.030^a^ (1.08)	10.96 ± 2.726^a^ (0.92)	10.80 ± 1.860^a^ (0.90)
* EBP1*	95.48 ± 13.552^a^ (1.05)	85.76 ± 20.561^a^ (0.95)	84.79 ± 13.540^a^ (0.92)
* ARGOS*	0.93 ± 0.183^a^ (1.23)	0.58 ± 0.126^a^ (0.77)	0.65 ± 0.122^a^ (0.83)
Chloroplast-targeted genes			
* CHLM*	1.97 ± 0.208^a^ (0.76)	3.24 ± 0.913^a^ (1.24)	2.83 ± 0.659^a^ (1.09)
* CHL27*	2.79 ± 0.531^a^ (0.47)	9.15 ± 3.558^a^ (1.53)	3.17 ± 0.59^a^ (0.53)
* PORB*	18.43 ± 0.372^a^ (0.96)	20.05 ± 0.210^a^ (1.04)	18.31 ± 0.276^a^ (0.95)
* LHCA2*	428.10 ± 155.529^a^ (0.96)	449.70 ± 131.546^a^ (1.02)	574.12 ± 318.830^a^ (1.31)
* PORC*	7.60 ± 4.034^a^ (0.87)	9.77 ± 2.704^a^ (1.13)	11.92 ± 5.452^a^ (1.37)
* PsbS*	0.03 ± 0.017^a^ (1.26)	0.02 ± 0.006^a^ (0.74)	0.01 ± 0.003^a^ (0.56)
* ATPD*	122.09 ± 5.773^b^ (0.86)	163.22 ± 13.719^b^ (1.14)	91.23 ± 9.503^a^ (0.64*)
* PsbP*	718.75 ± 75.843^a^ (1.03)	682.77 ± 44.241^a^ (0.97)	612.86 ± 49.441^a^ (0.87)
	5 DAS		
	S27 (female)	R29 (male)	F_1_-S27 × R29
Genes involved in organ size			
* ANT*	0.03 ± 0.004^b^ (1.23)	0.02 ± 0.002^a^ (0.82)^b^	0.02 ± 0.002^a^ (0.62*)
* CYCD3;1*	6.82 ± 0.801^b^ (1.21)	4.49 ± 0.470^a^ (0.79)	4.70 ± 0.671^a^ (0.83)
* EBP1*	33.02 ± 3.196^a^ (1.14)	24.89 ± 4.347^a^ (0.86)	27.02 ± 3.787^a^ (0.93)
* ARGOS*	0.46 ± 0.088^a^ (1.37)	0.21 ± 0.038^a^ (0.63)	0.22 ± 0.021^a^ (0.66)
Chloroplast-targeted genes			
* CHLM*	2.20 ± 0.289^a^ (1.14)	1.67 ± 0.309^a^ (0.86)	1.79 ± 0.319^a^ (0.93)
* CHL27*	1.96 ± 0.168^a^ (1.13)	1.52 ± 0.389^a^ (0.87)	1.52 ± 0.340^a^ (0.88)
* PORB*	2.23 ± 0.169^a^ (0.97)	2.39 ± 0.340^a^ (1.03)	2.24 ± 0.313^a^ (0.97)
* LHCA2*	39.10 ± 5.653^a^ (1.08)	33.21 ± 5.320^a^ (0.92)	36.19 ± 5.032^a^ (1.00)
* PORC*	0.61 ± 0.078^a^ (0.77)	0.97 ± 0.245^a^ (1.23)	0.86 ± 0.165^a^ (1.09)
* PsbS*	0.00 ± 0.002^a^ (1.26)	0.00 ± 0.000^a^ (0.74)	0.00 ± 0.000^a^ (0.74)
* ATPD*	21.62 ± 0.925^a^ (1.05)	19.51 ± 1.521^a^ (0.95)	21.96 ± 2.212^a^ (1.07)
* PsbP*	153.34 ± 8.824^a^ (1.11)	122.08 ± 4.314^a^ (0.89)	171.79 ± 23.599^a^ (1.25)
	6 DAS		
	S27 (female)	R29 (male)	F_1_-S27 × R29
Genes involved in organ size			
* ANT*	0.01 ± 0.006^a^ (0.77)	0.01 ± 0.010^a^ (1.23)	0.00 ± 0.001^a^ (0.28)
* CYCD3;1*	2.10 ± 0.388^a^ (0.82)	3.02 ± 0.392^a^ (1.18)	2.57 ± 0.787^a^ (1.23)
* EBP1*	19.52 ± 3.345^a^ (0.77)	31.08 ± 4.424^a^ (1.23)	27.88 ± 8.320^a^ (1.10)
* ARGOS*	0.25 ± 0.067^a^ (1.07)	0.21 ± 0.026^a^ (0.93)	0.23 ± 0.035^a^ (1.01)
Chloroplast-targeted genes			
* CHLM*	4.15 ± 0.187^a^ (1.12)	3.24 ± 0.504^a^ (0.88)	3.68 ± 0.536^a^ (1.00)
* CHL27*	5.28 ± 0.742^a^ (1.13)	4.06 ± 0.369^a^ (0.87)	4.10 ± 0.257^a^ (0.88)
* PORB*	1.91 ± 0.060^ab^ (1.15)	1.41 ± 0.073^a^ (0.85)	3.37 ± 0.768^b^ (2.03**)
* LHCA2*	39.94 ± 2.660^a^ (1.06)	35.17 ± 3.957^a^ (0.94)	44.60 ± 1.578^a^ (1.19)
* PORC*	1.14 ± 0.324^a^ (0.68)	2.20 ± 0.525^a^ (1.32)	1.26 ± 0.331^a^ (0.76)
* PsbS*	0.01 ± 0.001^a^ (0.67)	0.01 ± 0.007^a^ (1.33)	0.01 ± 0.002^a^ (0.62)
* ATPD*	38.36 ± 7.410^a^ (0.93)	44.09 ± 9.913^a^ (1.07)	41.11 ± 15.227^a^ (1.00)
* PsbP*	192.31 ± 32.822^a^ (1.19)	131.54 ± 7.282^a^ (0.81)	216.30 ± 21.144^a^ (1.34)

Letters indicate significant differences at *p* < 0.05 (Student’s t-test)

The relative ratio of expression level compared with MPV (mid parent values) is shown in parentheses

*,*p* < 0.05 (F_1_ vs. MPV); **,*p* < 0.01 (F_1_ vs. MPV)

Mean ± Standard errors

### Chloroplast-targeted genes have increased expression levels in early developmental stages

We measured the expression level of eight genes involved in chlorophyll biosynthesis or in the photosynthesis process with products active in the chloroplast or plastid. At 2 DAS the expression levels of all eight genes were low in the F_1_ hybrids and parents, but were higher in the F_1_ hybrid than in parental lines (Fig. 3c, d, Table 4); 6 of the 8 genes, *ATPD*, *CHL27, CHLM*, *LHCA2*, *PORC*, *PsbP*, were significantly upregulated in the F_1_ hybrids relative to the MPV. At 3 DAS the expression of these chloroplast-targeted genes was increased in both F_1_ hybrids and parental lines, and only *LHCA2* had higher expression in the F_1_ hybrids than in the parental lines (Fig. 3c, d, Table 4). At 4 DAS there was a decrease in expression level of all eight genes in both F_1_ hybrids and parental lines, and the expression levels were similar in all lines (Fig. 3c, d, Table 4). At 5 and 6 DAS there was similar expression to the 4 DAS expression levels with no difference between the F_1_ hybrids and MPV except for *PORB* at 6 DAS (Fig. 3c, d, Table 4).

We examined the chlorophyll content per gram fresh weight at 6 DAS. The chlorophyll content of the F_1_ hybrid (0.136 ± 0.005 μg/mg) is similar to that of R29 (0.127 ± 0.010 μg/mg) but greater than that of S27 (0.094 ± 0.009 μg/mg). When the larger leaves are considered the total chlorophyll content of the F_1_ hybrid is greater than that of parents because of the increased size and number of cells resulting in an increased leaf area and fresh weight in the F_1_ hybrid.

### Transcriptome analysis of 2 DAS cotyledons

**Fig. 4:**
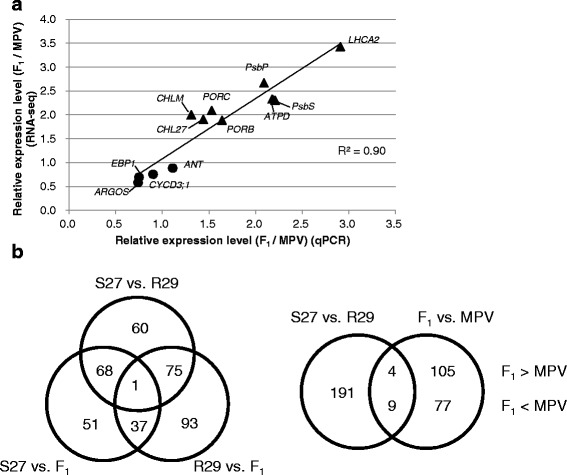
Verification of RNA-seq data by quantitative RT-PCR (**a**). Venn diagram representing the number of differentially expressed genes at 2 DAS (**b**). Filled circles and triangles in the scatter diagram show the organ size and chloroplast-targeted genes, respectively. MPV, mid parent value

As the expression levels of chloroplast-targeted genes tended to be higher in the F_1_ hybrids than MPV at 2 DAS (Table 4), we performed a transcriptome analysis in the parental lines (S27 and R29) and the F_1_ hybrid. To verify the RNA-seq analysis, we compared the relative ratio of expression levels between the F_1_ hybrid and MPV calculated by qPCR and RNA-seq data in the organ size-associated and chloroplast-targeted genes (Table 4, Additional file 2: Table S3). A high correlation (*r* = 0.95) was observed between the two analyses (Fig. 4a).

Less than 1 % of the genes showed a two-fold difference (log2 ratio > = 1.0) in expression with 95 % confidence between parental lines (204 of 41,174 genes) or between the F_1_ hybrid and each parental line (F_1_ vs. S27; 157 genes, F_1_ vs. R29; 206 genes) (Fig. 4b, Additional file 2: Tables S4-6). Between F_1_ hybrid and MPV (see Methods) 195 (0.5 %) genes showed a two-fold difference (log2 ratio > = 1.0) in expression with 95 % confidence, and 13 of these 195 genes were differential expressed in the parental lines (Fig. 4b, Additional file 2: Table S7).

We performed a Gene Ontology (GO) analysis of genes differentially expressed in the parental lines (S27 vs. R29), between the F_1_ hybrid and each parental line (F_1_ vs. S27, F_1_ vs. R29), and between the F_1_ hybrid and the MPV (Table 5, Additional file 2: Tables S8-S11). In the upregulated genes in the F_1_ hybrid compared with S27, R29, or MPV, GO categories of ‘Photosynthesis’ and ‘Chloroplast part’ were overrepresented. In the downregulated genes in the F_1_ hybrid, the GO categories of ‘Response to heat’, ‘Response to high light intensity’, and ‘Response to temperature stimulus’ were over-represented (Table 5, Additional file 2: Tables S8-S11).

**Table 5:**
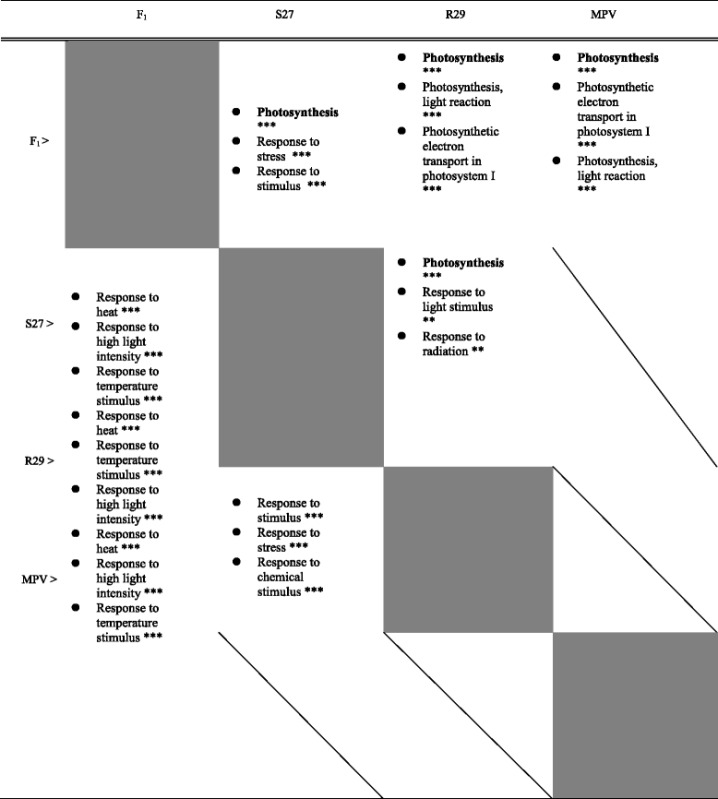
Top 3 of overrepresented GO terms in Biological process in differentially expressed genes among S27, R29, F_1_, and MPV

Expression levels in vertical on the left lines are higher than that in right of the horizontal lines

***,*P* < 0.001; **,*P* < 0.01

Overall, chloroplast-targeted genes, especially those having a function in photosynthesis, such as *Light harvesting chlorophyll a/b-binding protein* (*LHCB*), *Photosystem I subunit* (*PSA*), and *NDH-dependent Cyclic Electron Flow* (*NDF*) had a higher expression level in the F_1_ hybrid than in the parental lines and genes involved in the category of ‘response to heat’, ‘response to temperature stimulus’, and ‘response to high light intensity’ such as *Heat shock protein* (*HSP*) and *Heat stress transcription factor* (*HSF*) had a lower expression level in the F_1_ hybrid than in parental lines (Additional file 1: Fig. S3, Table 5, Additional file 2: Tables S5-S7, S9-S11).

### Identification of allele specific expressed genes in the F_1_ hybrid

**Fig. 5:**
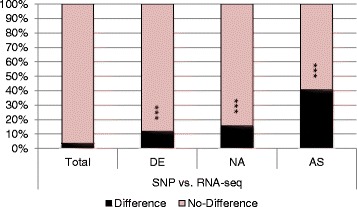
Bar graph of the percentage of the genes showing different allelic expression ratios to their parental expression levels (between AEL (SNP) and REL (RNA-seq)) (*p* < 0.01). Total, all expressed genes (except for non-expressed genes in S27 and/or R29); DE, differentially expressed genes between parental lines; NA, non-additively expressed genes between F_1_ hybrid and mid parent value; AS, allele-specific expressed genes in F_1_ hybrid satisfying the following criterion, five fold difference of SNP numbers per site between parental alleles (*p* < 0.05) or *p* < 0.001 if only one-parent SNP was detected. ***,*p* < 0.0001

The parental alleles expressed in the F_1_ hybrid were identified through a SNP analysis. The two allelic expression levels in each gene in the F_1_ hybrid (AEL) were compared to the relative expression levels (REL) in the two parents. 436 (3.5 %) of 12,321 (excluding 449 non-expressed genes in S27 and/or R29) genes showed a difference between AEL and REL (*p* < 0.01) (Fig. 5, Additional file 1: Fig. S4). Genes that were either differentially expressed between the parents (11.9 %) or showed differential expression relative to the MPV (15.8 %) were overrepresented (Fig. 5, Additional file 1: Fig. S5).

We identified allele-specific expressed genes in the F_1_ hybrid. We classified genes as allele-specific expressed if they satisfied the following criterion: five fold difference of SNP numbers per site between S27 and R29 alleles (*p* < 0.05) or *p* < 0.001 if only one-parental SNP was detected. We found 162 (41; only S27 alleles, 121; S27 > R29) S27 allele specific and 194 (39; only R29 alleles, 155; R29 > S27) R29 allele specific genes (Additional file 1: Fig. S6, Additional file 2: Table S12). 145 (40.7 %) of 356 allele-specific expressed genes showed a difference between AEL and REL (Fig. 5).

We performed a GO analysis of these allele specific genes. In the S27 allele specific expressed genes, GO categories of ‘Cytoplasm’, ‘Chloroplast’, ‘Ribosome’, and ‘Translation’ showed significant enrichment (Additional file 2: Table S13). In the R29 allele specific expressed genes, GO categories of ‘Cytoplasm’, ‘Ribosome’, ‘Response to water’, and ‘Translation’ showed significant enrichment (Additional file 2: Table S13). Genes categorized into both ‘Translation’ and ‘Ribosome’ tended to show both S27 and R29-allele specific expression in the F_1_ hybrid (Additional file 1: Fig. S7).

### Shutdown of chlorophyll biosynthesis in the cotyledon decreased heterosis

Chloroplast-targeted genes were upregulated in the F_1_ hybrid at 2 DAS, especially those having a function in photosynthesis. To examine the relationship between photosynthesis and increased cotyledon/leaf area at an early developmental stage, young seedlings were treated with norflurazon, an inhibitor of phytoene desaturase, at two different stages [23]. Seeds were grown on MS medium for one week, and transferred to MS medium with 1.0 μM norflurazon and grown a further two weeks. The treated seedlings did not produce chlorophyll and had white 1st and 2nd leaves (Additional file 1: Fig. S8). The 1st and 2nd leaves of the F_1_ hybrids were larger than those of parental lines after two weeks on the norflurazon medium (Table 6, Additional file 1: Fig. S8). Seeds grown on MS medium with 1.0 μM norflurazon for one week and transferred to MS medium without norflurazon did not show any heterosis (Table 6, Additional file 1: Fig. S8), though plants did recover chlorophyll biosynthesis after removal of norflurazon as reported [24]. These experiments show that photosynthesis at the cotyledon stage is critical for heterosis in the F_1_ hybrid.

**Table 6:** Leaf area after norflurazon treatment

	R29 (male)	S27 (female)	F_1_-S27 × R29	Relative to MPV	Relative to BPV
Relative ratio in leaf area compared with R29					
A. Leaf area in 1st and 2nd leaves after three weeks sowing	1.00^a^	1.19 ± 0.15^a^	2.74 ± 0.33^b^	2.54	2.29
B. Leaf area in 3rd and 4th leaves after four weeks sowing	1.00^a^	1.92 ± 0.17^b^	1.50 ± 0.18^ab^	0.93	0.78

A. Seeds were sown on MS medium and grown for one week. The seedlings were transferred to MS medium with 1.0 μM norflurazon and grown for a further two weeks

B. Seeds were grown on MS medium with 1.0 μM norflurazon for one week, then transferred to MS medium for three weeks

Letters (a and b) indicate significant differences at *p* < 0.05 (Student’s t-test)

*MPV* mid-parent values, *BPH* best-parent values

Mean ± Standard errors

## Discussion

### Heterosis is observed in mature seeds, post-germination seedlings, and mature plants

The pattern of development showing different aspects of heterosis in Chinese cabbage is similar to that described for *A. thaliana*, another member of the Brassica family [9–11, 25–27]. We showed that the mature seed of the F_1_ hybrid is larger than the seeds of either of the parents, and the area of the embryo is greater in the F_1_ hybrid than in the parents. Large embryo sizes and increased post germination seedling sizes have been reported in *A. thaliana* and maize F_1_ hybrids [9–11, 26, 28, 29], suggesting that the seed heterosis in *B. rapa* is likely to be an innate characteristic of the F_1_ hybrid rather than a result of the sodium chloride treatment in parents used to overcome the self-incompatibility (see Methods).

In *A. thaliana*, the larger size of the cotyledon and leaves of F_1_ hybrids are associated with increased size and number of the photosynthetic palisade mesophyll cells. At maturity, the C24 x Col hybrid has approximately 25 %–30 % greater biomass than either of the parents [9, 10]. In “W39”, the R29 male parent has larger photosynthetic cells than the S27 female parent, which has an increased cell number relative to R29, and the F_1_ hybrid combines both these properties. Difference in cell number or size did not result in difference in the organ size between parental lines, but the increased cell number and size in the F_1_ hybrid resulted in an increased organ size and was associated with an increased photosynthetic capacity. Heterotic F_1_ hybrids of *A. thaliana* also showed both increased cell number and size [9–11], suggesting that the occurrence of both events is important for increased organ size in heterotic F_1_ hybrids. We checked whether the increased cell size could be attributed to endopolyploidy and found that there was no difference in the distribution of cell ploidies in the F_1_ hybrids and parents. Since it is known that increased cell size or number in the leaves of plants is correlated with increased chloroplast number and chlorophyll content, it is likely that the overall amount of photosynthesis in the hybrid plant is greater than in either of the parents [9, 30]. As the leaves of the hybrid “W39” are greater in total area than the parents and the chlorophyll content per fresh weight in “W39” was similar to that of best parent, an increased production of photosynthate could be expected.

Plant hormones play important roles in regulating plant growth and development. We measure the hormone levels in 2 day cotyledons and 10 day leaves, before or just after the appearance of the increased leaf area. Most of the hormone concentrations in the F_1_ hybrid were within the parental range. As sensitivity to hormone signalling is important for the heterosis phenotype in *A. thaliana* [19], sensitivity rather than concentrations of hormones may be important for the heterosis phenotype.

Tissue, organ or stage-specific heterosis has been observed in a number of plants and these all result in increased yield [31, 32]. Heterosis in the “W39” F_1_ hybrid of Chinese cabbage results in a greater harvestable biomass than in the parents. It is possible that changes in the leaf cells in some of the earliest stages of the germinating seedling may lead to the continuing increase in size of leaves in the F_1_ hybrid with genetic factors responsible for increased cell number and size. This property could be of fundamental importance in generating the increased biomass of the F_1_ hybrid. Further study will be required to determine whether increased cotyledon or leaf size is a general predictor of high yield heterosis in *B. rapa* F_1_ hybrids.

### Chloroplast-targeted genes were upregulated in F_1_ hybrids at two days after sowing

There are reports which claim to identify heterosis related genes such as a flowering time gene in tomato, circadian rhythm genes in *A. thaliana*, and organ size genes in maize and *Larix* [20, 21, 33, 34]. We examined four genes whose orthologs in *A. thaliana* were involved in leaf size control. In S27, which has more cells than R29 and F_1_ hybrids, the expression level of the three genes, *CYCD3;1*, *EBP1*, and *ARGOS*, was higher than that in R29 and F_1_ hybrid at 2–3 DAS, and these three genes are similarly expressed in the F_1_ hybrid and R29. Though increased cell number in S27 is related to the increased expression levels of these three genes, the increased cotyledon size in the F_1_ hybrid, being partly dependent on increased cell number, is less dependent on the pathway involving these genes.

Upregulation of chloroplast-targeted genes occurs in the Arabidopsis C24 x Col hybrid, the heterotic intra-specific hybrids of rice, and the heterotic inter-specific hybrids of *A. thaliana* and related species [9, 33, 35, 36]. Eight of the upregulated chloroplast-targeted genes reported in the Arabidopsis C24 x Col hybrid were upregulated in “W39” at 2 DAS. The 2 DAS transcriptome analysis identified genes involved in the categories of ‘Photosynthesis’ and ‘Chloroplast part’ as upregulated in the F_1_ hybrid compared to the parental lines. This transient increase in gene expression of the photosynthesis related genes on day 2 may be a prerequisite to the continuing increases in both cell size and number of photosynthetic cells, processes initiated in the cotyledon in the final growth stages of the seed. A dependence on photosynthesis in the cotyledon stage for subsequent heterosis in germinating seedlings was suggested by the results of the norflurazon treatment on young seedlings. Plants could grow during the one-week norflurazon treatments of seeds because sucrose was provided by the medium. However equalizing the source by blocking photosynthesis eliminates the heterosis phenotype even when plants are grown on MS medium for 2 weeks after norflurazon treatment. This suggests that an increased production of photosynthesis in the first week of cotyledon growth is important for increased leaf size in F_1_ hybrids even after the cotyledon stage. The transient increase in gene expression of the photosynthesis related genes in the cotyledon may be required for the heterosis seen after the cotyledon stage.

### Genes involved in stress were downregulated in F_1_ hybrids at two days after sowing

In C24 x Col hybrids in *A. thaliana*, genes in the stress response category were overrepresented in both up- and downregulated genes in the F_1_ hybrid relative to the MPV at both cotyledon and seedling stages [9]. Differential expression of stress responsive genes between inter- or intra-specific hybrids and their parental lines has been widely observed in plants [9–11, 36, 37]. In this study, we found downregulation of genes involved in the categories of ‘Response to heat’, ‘Response to temperature stimulus’, and ‘Response to high light intensity’ such as *HSP* genes in the F_1_ hybrid relative to parental lines. It is not clear whether this implies that the F_1_ hybrid may be less responsive to environmental effects or it is only obvious in unchallenged conditions. Downregulation of *HSP* genes was also observed in heterotic inter-specific hybrids between *A. thaliana* and *A. arenosa*, and the authors suggested this is due to buffering effects [37], which may be involved in the vigour phenotype.

### Allele-specific expressed genes

RNA-seq enables us to distinguish the parental alleles of transcripts in F_1_ hybrids at the whole genome level. In this study, we compared the AEL and REL in all expressed genes, genes differentially expressed between parental lines, or genes non-additively expressed between F_1_ hybrid and MPV. Fewer than 16 % of genes showed a significant difference between AEL and REL, suggesting that differences in the expression levels between parental lines is maintained in the allelic bias of transcripts in F_1_ hybrids. Of the AEL genes, about 45.7 % of genes had more transcripts derived from S27 alleles than that from R29 alleles, indicating that there is no preference for the expression alleles from one parent in the F_1_ transcripts.

We identified 365 genes as being allele-specific expressed, and the GO categories of ‘Translation’ and ‘Ribosome’ were over-represented in both S27- and R29-allele specific expressed genes. Mutations in ribosomal protein genes in *A. thaliana* cause various types of developmental defects including in leaf development and cell proliferation [38, 39]. Single recessive mutants of the ribosomal protein genes, *api2/rpl36ab* or *rpl36aa*, showed a pointed-leaf phenotype, and these two genes with identical amino acid sequences are located on different chromosomes. The hybrid between *apl2* and *rpl36aa* (*API2/api2*; *RPL36aA/apl36aa*) revealed the same phenotype as each of the single mutants, indicating that non-allelic non-complementation of ribosomal proteins combining to produce haploinsufficiency, plays a role in leaf development [40]. Different combinations of ribosomal proteins caused by allele-specific expression of ribosomal proteins observed in this study may be related to the increased leaf area in F_1_ hybrid.

## Conclusions

The heterosis phenotype first seen in the cotyledons was observed a few days after sowing. Most genes showed an additive expression pattern, and any difference of expression levels between parental lines was maintained in the F_1_ hybrids. Genes categorized in the GO analysis into ‘Photosynthesis’ and ‘Chloroplast part’ tended to be upregulated in F_1_ hybrids at 2 DAS. Norflurazon treatment on germinating seeds leads to a white cotyledon and reduced heterosis in leaves. Norflurazon treatment on one-week seedlings, which have green cotyledons, continued to have heterosis in leaf size. These observations suggest the upregulation of chloroplast-targeted genes in the cotyledon and photosynthesis at the cotyledon stage are important for increased leaf area in F_1_ hybrids, and this increased leaf area could lead to the increased yield seen at harvest.

### Availability of supporting data

All supporting data are included as additional files. The RNA sequencing data have been deposited with DDBJ under DRA003125.

## Additional files

Additional file 1: Figure S1. Development of S27, R29, and F_1_ hybrid. (A) Two day seedlings of S27, R29, and F_1_ hybrid. The number of true leaves (B) and fresh weight at 30 DAS (C) in F_1_ hybrid and parental lines. **Figure S2.** Flow cytometry analysis of nuclei from cotyledon at 6 DAS (A) and 1st and 2nd leaves at 14 DAS (B) in S27, R29, and the F_1_ hybrid. **Figure S3.** Bar graph of the expression levels of upregulated (left panel) and downregulated (right panel) genes in F_1_ hybrid compared with parental lines. **Figure S4.** Comparison between relative ratio of SNP numbers between parental alleles in F_1_ hybrid (x axis) and relative expression levels between parental lines (y axis) in the total expressed genes. **Figure S5.** Comparison between ratio of SNP numbers in parental alleles in F_1_ hybrid (x axis) and relative expression levels in parental lines (y axis) in the non-additively expressed genes between F_1_ and mid parent value (circles) and differentially expressed genes between parental lines (squares). **Figure S6.** Scatter diagram of SNP numbers of S27 alleles (x axis) and R29 alleles (y axis) in F_1_ hybrid transcripts. **Figure S7.** Parental allelic ratio in allele-specific expressed genes involved in the GO category of ‘Ribosome’. **Figure S8.** Phenotypes with norflurazon treatment. (PPT 11230 kb) Additional file 2: Table S1. Sequences of primers used for quantitative RT-PCR. **Table S2.** Plant hormone contents in 2 day cotyledon and 10 day 1st and 2nd leaves in S27, R29, F_1_. **Table S3.** Expression level of organ size associated and chloroplast-targeted genes by RNA-seq. **Table S4.** Differentially expressed genes between S27 and R29 in 2 day cotyledon. **Table S5.** Differentially expressed genes between S27 and F_1_ in 2 day cotyledon. **Table S6.** Differentially expressed genes between R29 and F_1_ in 2 day cotyledon. **Table S7.** Differentially expressed genes between F_1_ and MPV (Mid Parent Values) in 2 day cotyledon. **Table S8.** GO term overrepresented in differentially expressed genes between parental lines in 2 day cotyledon. **Table S9.** GO terms overrepresented in differentially expressed genes between S27 and F_1_ in 2 day cotyledon. **Table S10.** GO terms overrepresented in differentially expressed genes between R29 and F_1_ in 2 day cotyledon. **Table S11.** GO terms overrepresented in differentially expressed genes between F_1_ and MPV (Mid parent values) in 2 day cotyledon. **Table S12.** Allele-specific expressed genes in F_1_ hybrid. **Table S13.** GO term overrepresented in allele specific expressed genes in F_1_ at 2 days after sowing. (XLSX 157 kb)

## Abbreviations

AEL: Allelic expression level in the F_1_ hybrid
ANT: Aintegumenta
ARGOS: Auxin-regulated gene involved in organ size
ATPD: ATP synthase delta-subunit gene
CHLM: Magnesium-protoporphyrin IX methyltransferase
CYCD3: Cyclin D3
DAS: Day after sowing
EBP1: ErbB-3 epidermal growth factor receptor binding protein 1
GO: Gene ontology
LHCA2: Photosystem I light harvesting complex 2
MPV: Mid parent value
POR: Protochlorophylide oxidoreductase
PsbP: Photosystem II subunit P
REL: Relative expression level in the two parents
