# Supplementary material for: Molecular and cellular characteristics of hybrid vigour in a commercial hybrid of Chinese cabbage
Source: BMC Plant Biol. 2016 Feb 17;16:45. doi: 10.1186/s12870-016-0734-3 (PMC4756405; doi:10.1186/s12870-016-0734-3)
Supplement: Additional file 1: Figure S1. — Development of S27, R29, and F1 hybrid. (A) Two day seedlings of S27, R29, and F1 hybrid. The number of true leaves (B) and fresh weight at 30 DAS (C) in F1 hybrid and parental lines. Figure S2. Flow cytometry analysis of nuclei from cotyledon at 6 DAS (A) and 1st and 2nd leaves at 14 DAS (B) in S27, R29, and the F1 hybrid. Figure S3. Bar graph of the expression levels of upregulated (left panel) and downregulated (right panel) genes in F1 hybrid compared with parental lines. Figure S4. Comparison between relative ratio of SNP numbers between parental alleles in F1 hybrid (x axis) and relative expression levels between parental lines (y axis) in the total expressed genes. Figure S5. Comparison between ratio of SNP numbers in parental alleles in F1 hybrid (x axis) and relative expression levels in parental lines (y axis) in the non-additively expressed genes between F1 and mid parent value (circles) and differentially expressed genes between parental lines (squares). Figure S6. Scatter diagram of SNP numbers of S27 alleles (x axis) and R29 alleles (y axis) in F1 hybrid transcripts. Figure S7. Parental allelic ratio in allele-specific expressed genes involved in the GO category of ‘Ribosome’. Figure S8. Phenotypes with norflurazon treatment. (PPT 11230 kb) [file 12870_2016_734_MOESM1_ESM.ppt]

## Slide 1
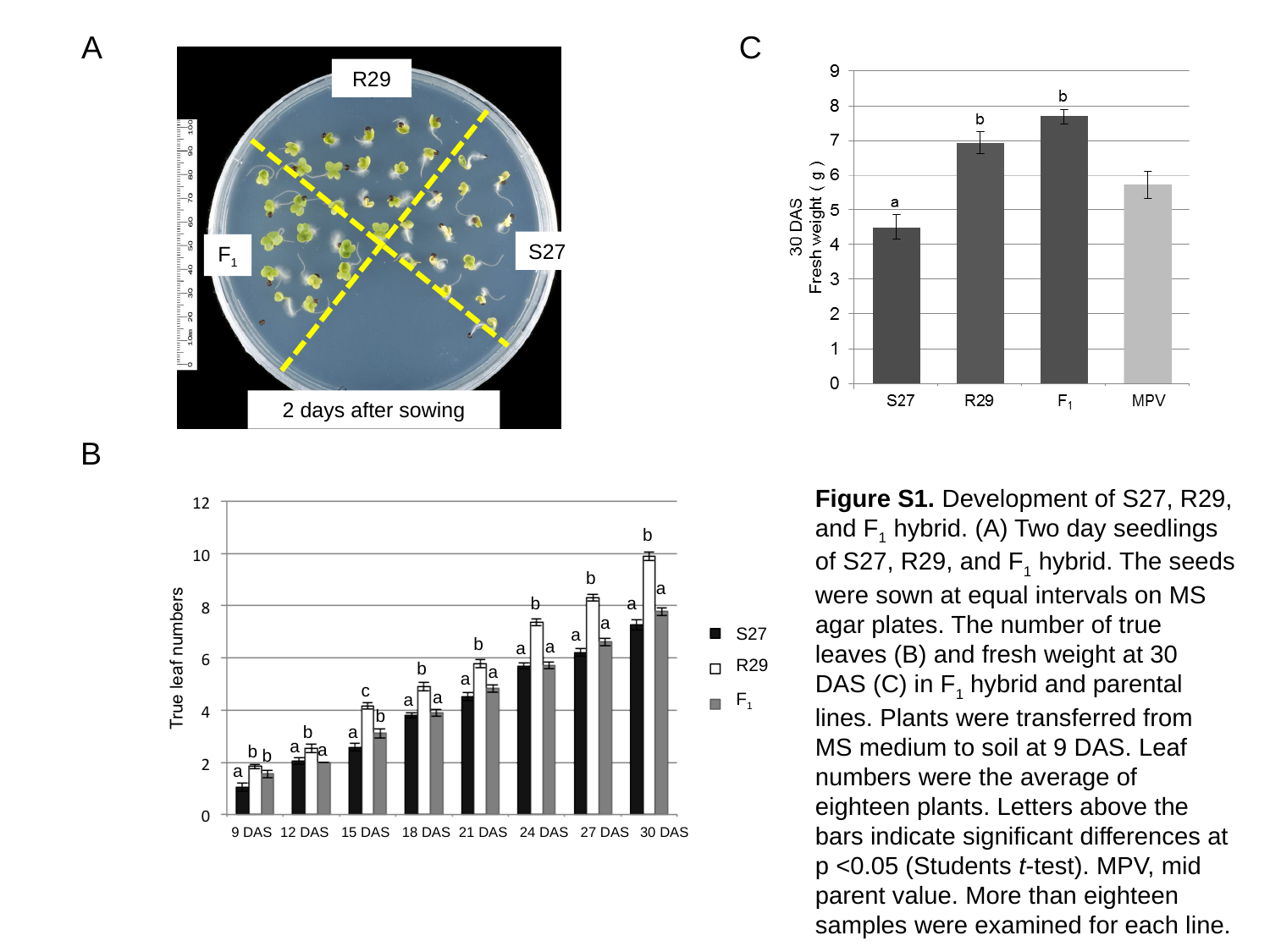

A
C
R29
S27
F1
2 days after sowing
B
Figure S1. Development of S27, R29, and F1 hybrid. (A) Two day seedlings of S27, R29, and F1 hybrid. The seeds were sown at equal intervals on MS agar plates. The number of true leaves (B) and fresh weight at 30 DAS (C) in F1 hybrid and parental lines. Plants were transferred from MS medium to soil at 9 DAS. Leaf numbers were the average of eighteen plants. Letters above the bars indicate significant differences at p <0.05 (Students t-test). MPV, mid parent value. More than eighteen samples were examined for each line.
S27
R29
F1
9 DAS 12 DAS 15 DAS 18 DAS 21 DAS 24 DAS 27 DAS 30 DAS
b
b
a
b
a
a
a
b
a
a
b
a
a
c
a
a
b
b
a
a
a
b
b
a

## Slide 2
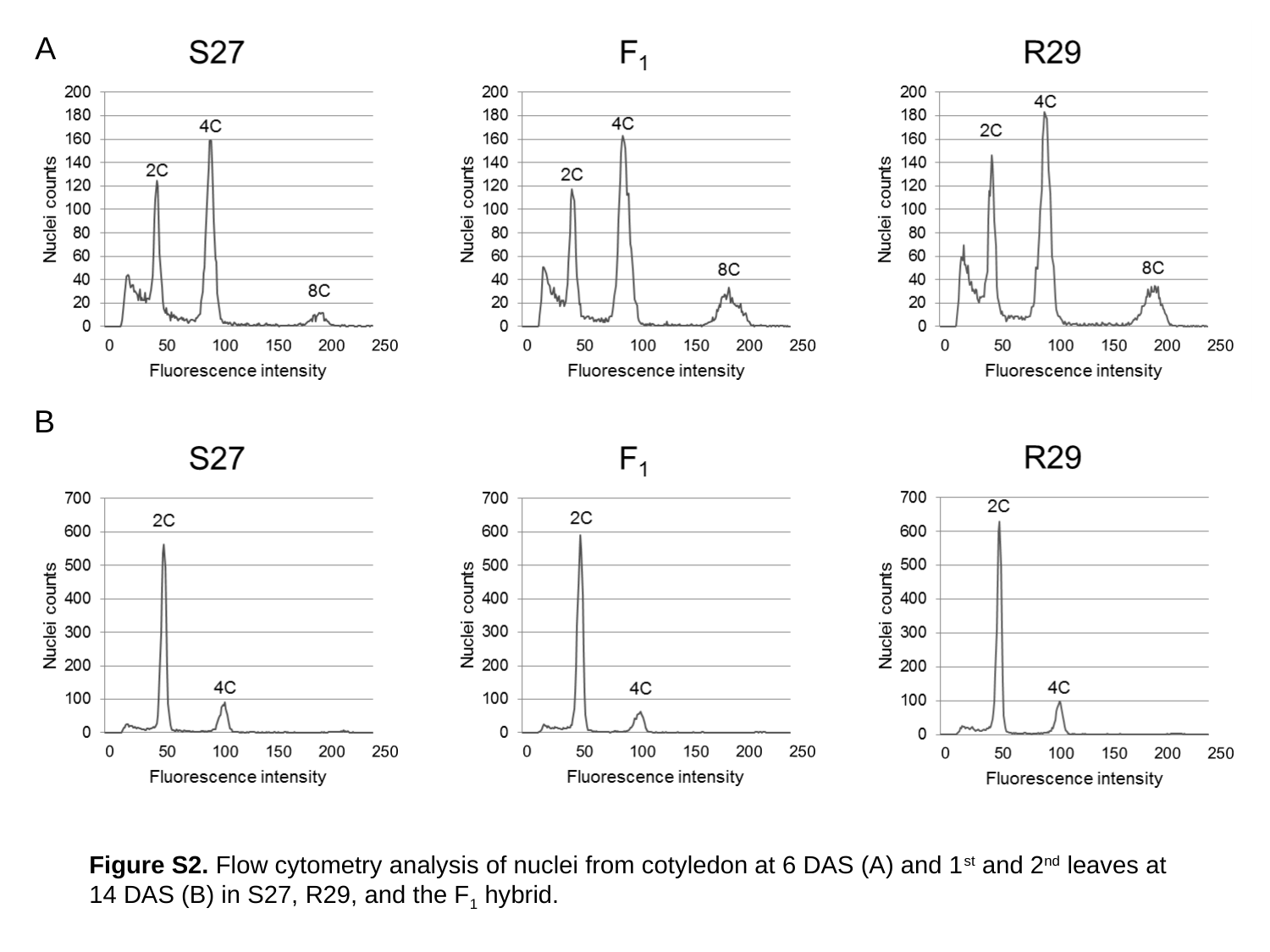

A
B
Figure S2. Flow cytometry analysis of nuclei from cotyledon at 6 DAS (A) and 1st and 2nd leaves at 14 DAS (B) in S27, R29, and the F1 hybrid.

## Slide 3
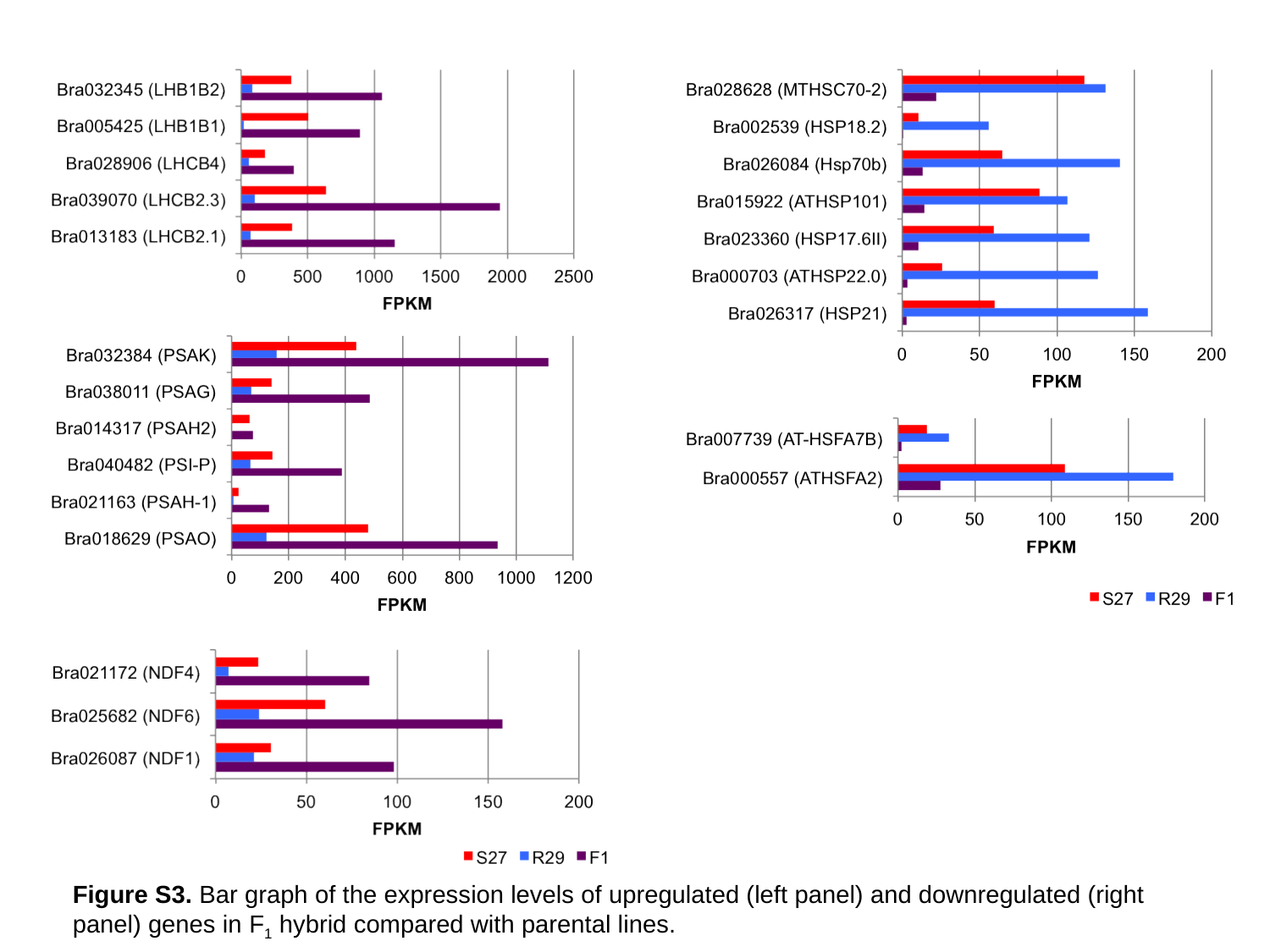

Figure S3. Bar graph of the expression levels of upregulated (left panel) and downregulated (right panel) genes in F1 hybrid compared with parental lines.

## Slide 4
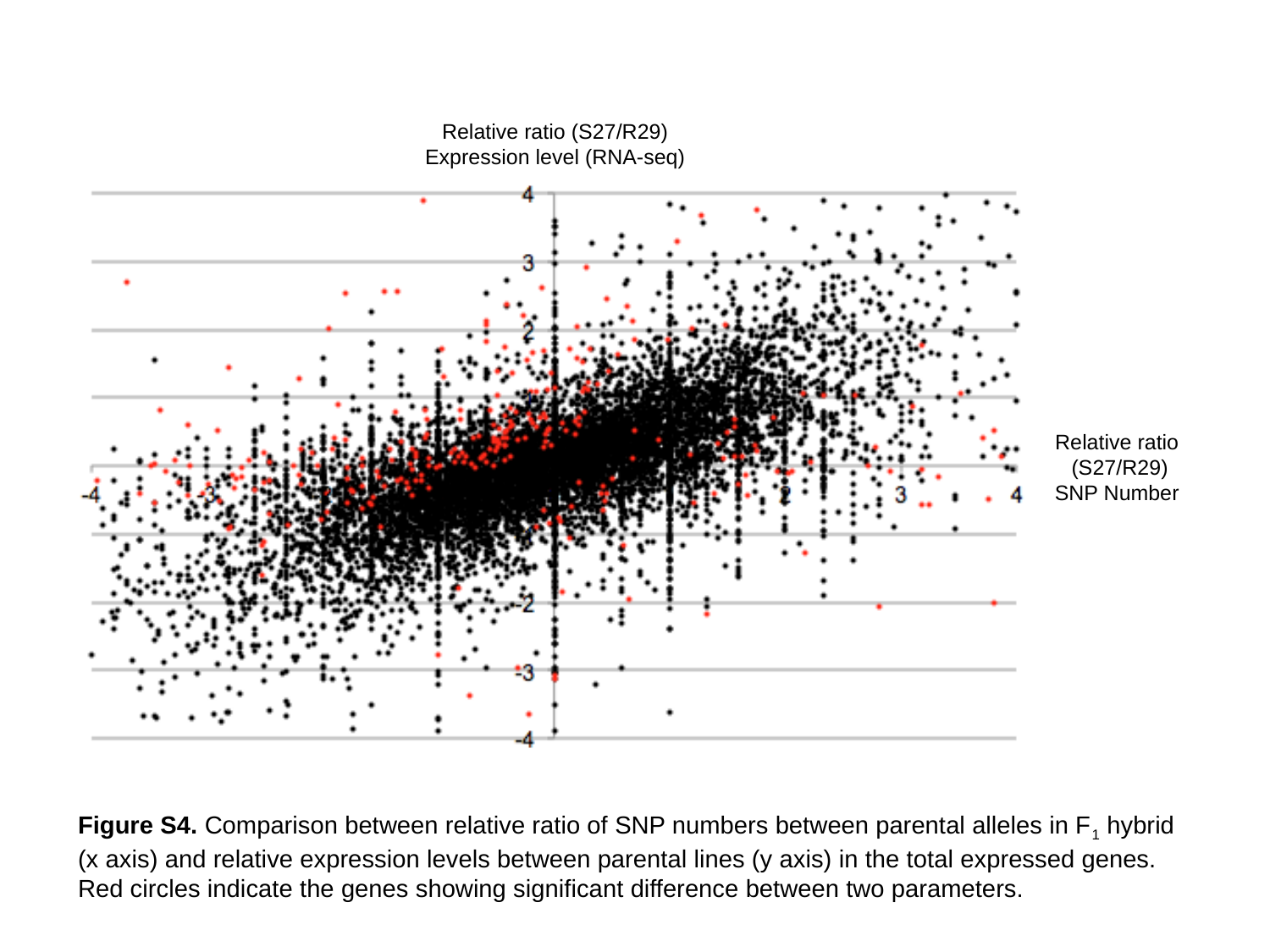

Relative ratio (S27/R29)
Expression level (RNA-seq)
Relative ratio
 (S27/R29)
SNP Number
Figure S4. Comparison between relative ratio of SNP numbers between parental alleles in F1 hybrid (x axis) and relative expression levels between parental lines (y axis) in the total expressed genes. Red circles indicate the genes showing significant difference between two parameters.

## Slide 5
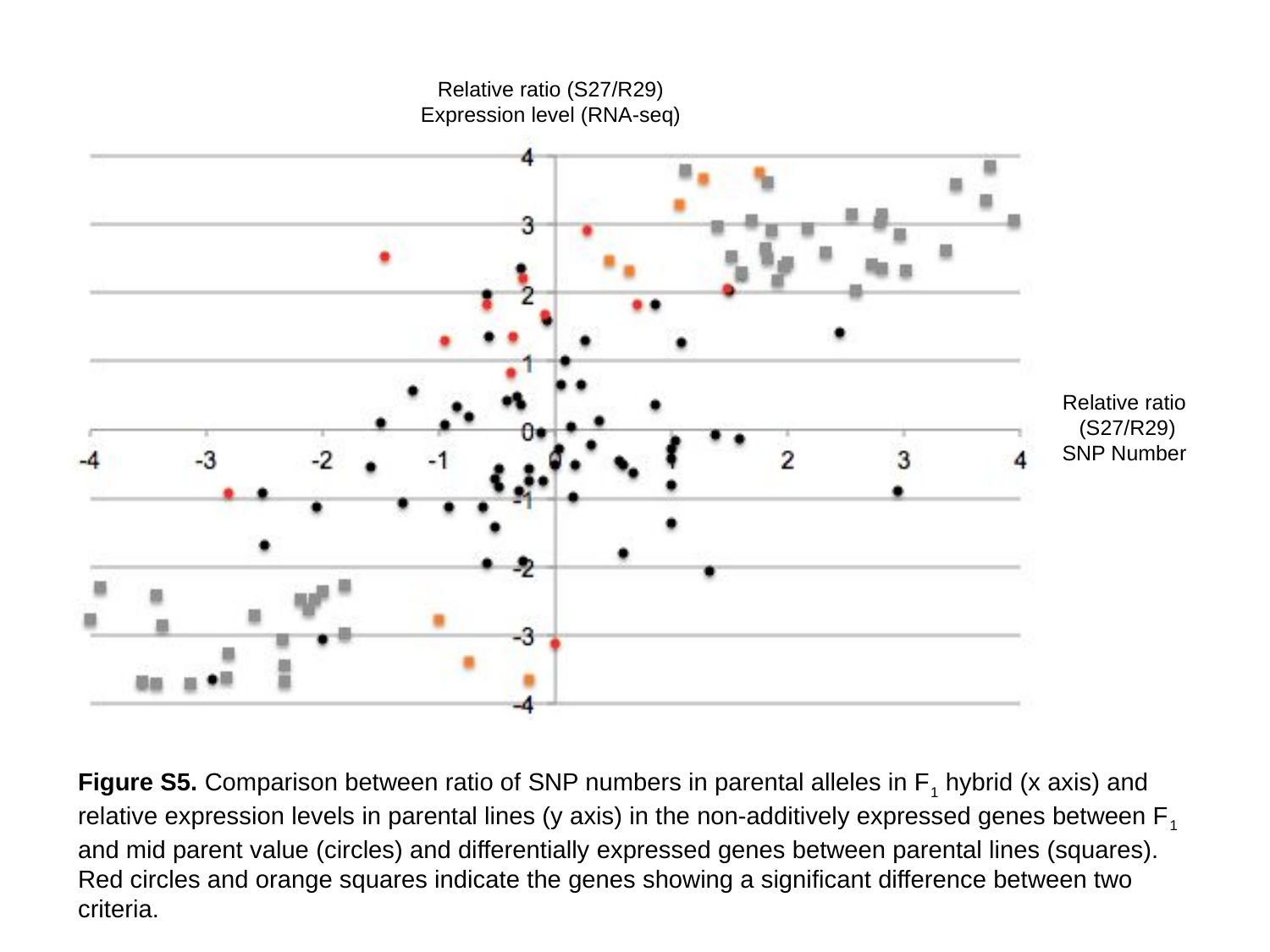

Relative ratio (S27/R29)
Expression level (RNA-seq)
Relative ratio
 (S27/R29)
SNP Number
Figure S5. Comparison between ratio of SNP numbers in parental alleles in F1 hybrid (x axis) and relative expression levels in parental lines (y axis) in the non-additively expressed genes between F1 and mid parent value (circles) and differentially expressed genes between parental lines (squares). Red circles and orange squares indicate the genes showing a significant difference between two criteria.

## Slide 6
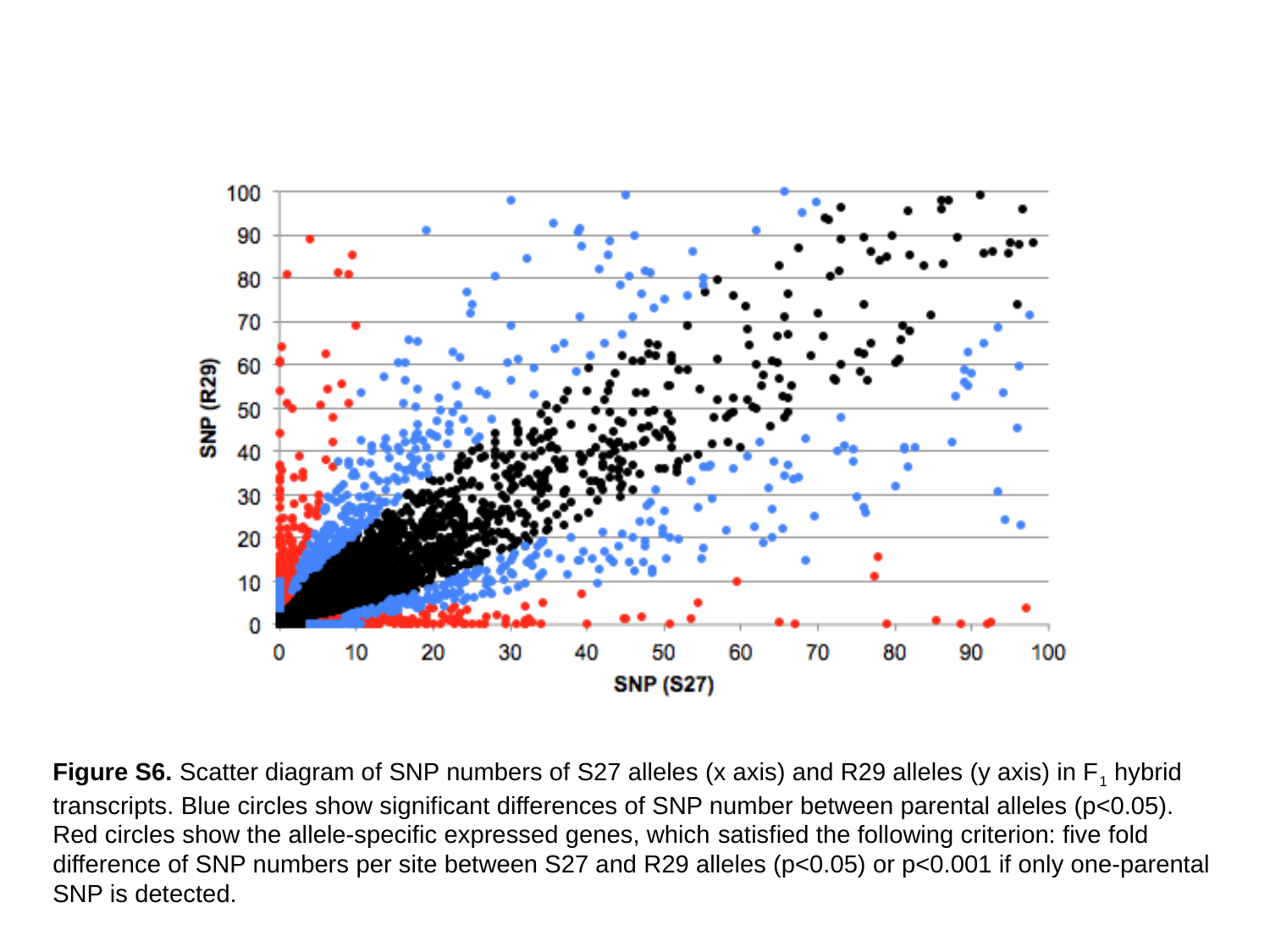

Figure S6. Scatter diagram of SNP numbers of S27 alleles (x axis) and R29 alleles (y axis) in F1 hybrid transcripts. Blue circles show significant differences of SNP number between parental alleles (p<0.05). Red circles show the allele-specific expressed genes, which satisfied the following criterion: five fold difference of SNP numbers per site between S27 and R29 alleles (p<0.05) or p<0.001 if only one-parental SNP is detected.

## Slide 7
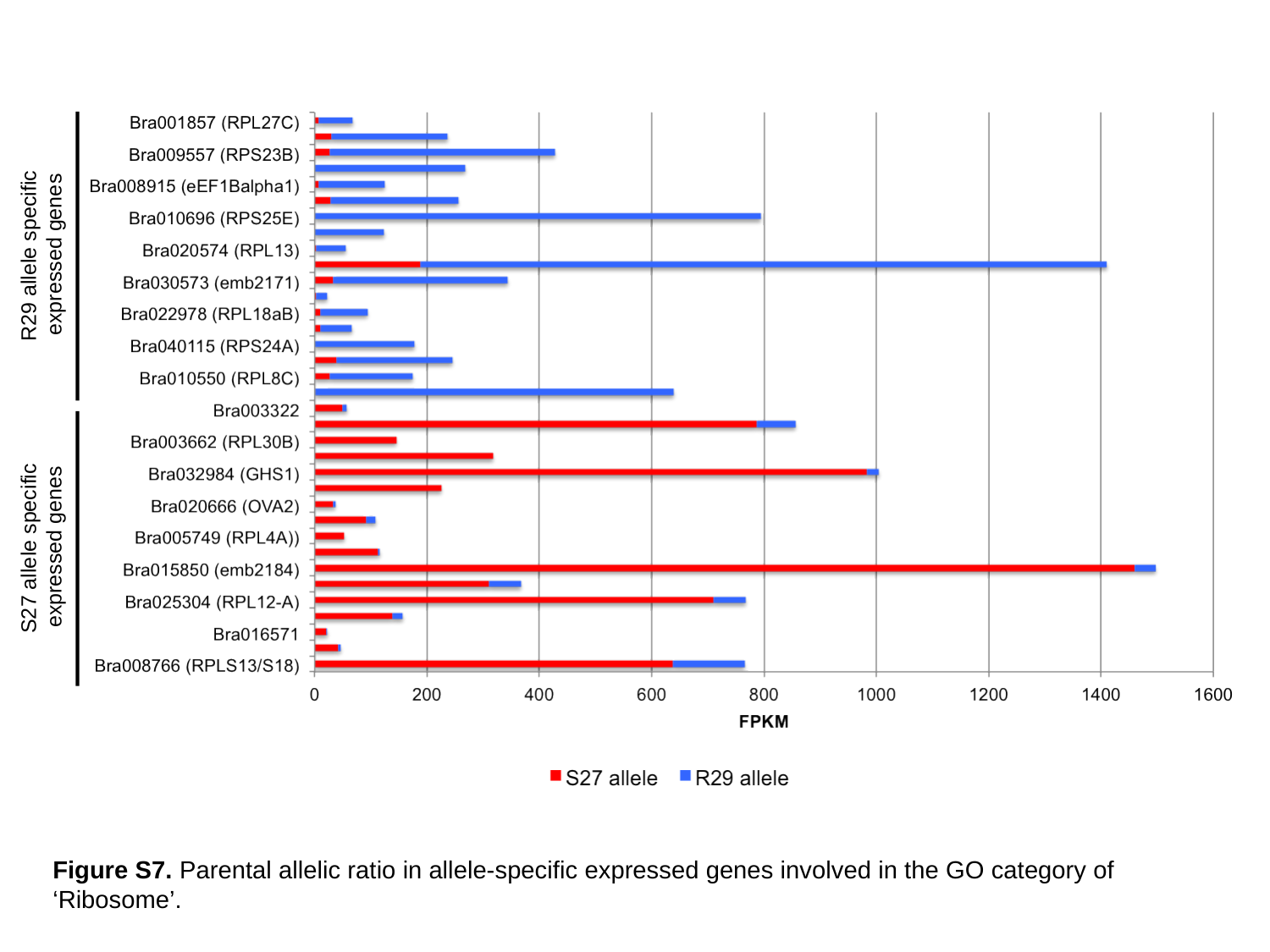

R29 allele specific
 expressed genes
S27 allele specific
 expressed genes
Figure S7. Parental allelic ratio in allele-specific expressed genes involved in the GO category of ‘Ribosome’.

## Slide 8
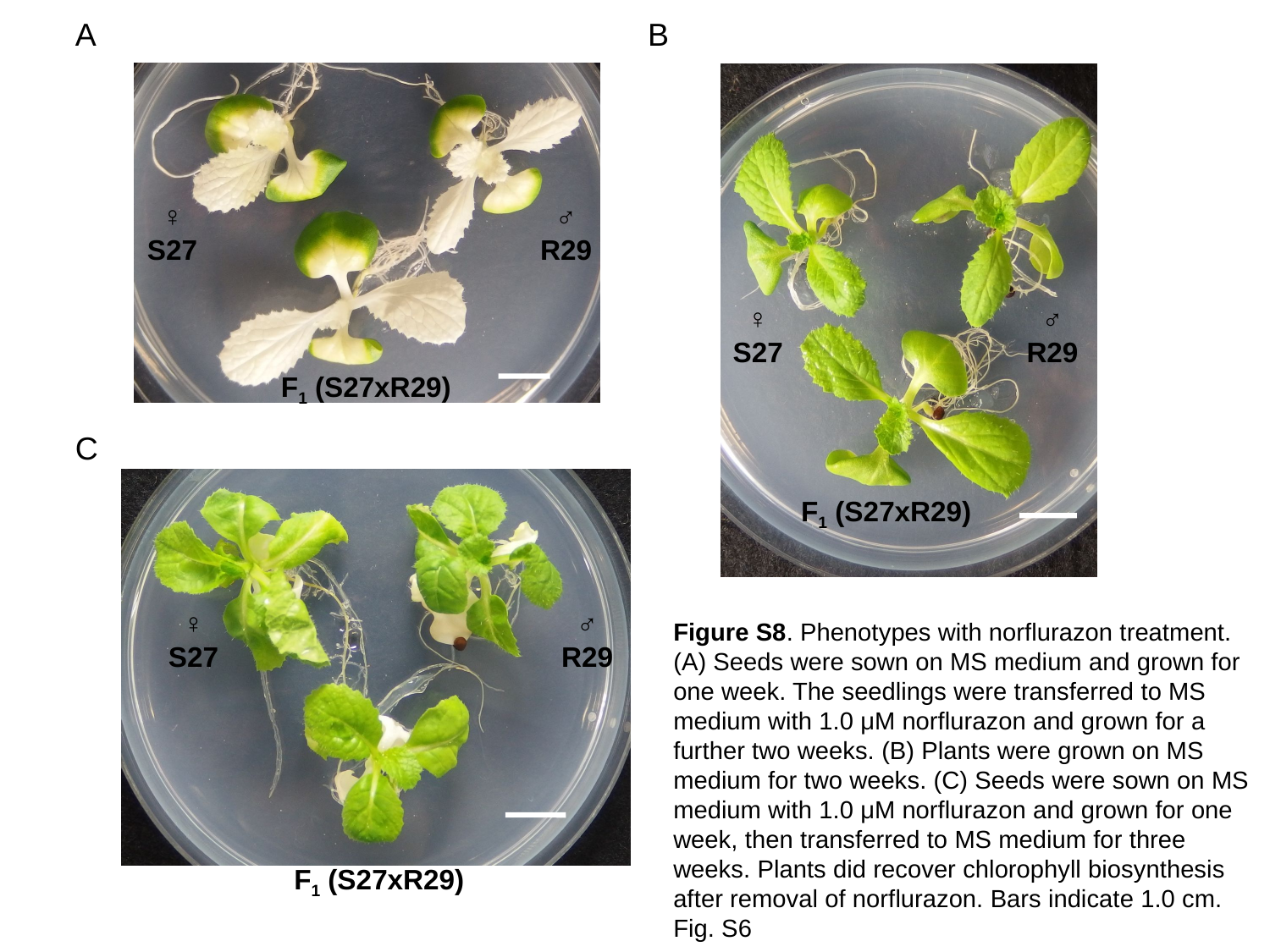

A
B
♀
S27
♂
R29
♀
S27
♂
R29
F1 (S27xR29)
C
F1 (S27xR29)
♀
S27
♂
R29
Figure S8. Phenotypes with norflurazon treatment. (A) Seeds were sown on MS medium and grown for one week. The seedlings were transferred to MS medium with 1.0 μM norflurazon and grown for a further two weeks. (B) Plants were grown on MS medium for two weeks. (C) Seeds were sown on MS medium with 1.0 μM norflurazon and grown for one week, then transferred to MS medium for three weeks. Plants did recover chlorophyll biosynthesis after removal of norflurazon. Bars indicate 1.0 cm.
Fig. S6
F1 (S27xR29)
